# Transcatheter Aortic Valve Replacement (TAVR) Versus Surgical Aortic Valve Replacement (SAVR): A Review on the Length of Stay, Cost, Comorbidities, and Procedural Complications

**DOI:** 10.7759/cureus.54435

**Published:** 2024-02-19

**Authors:** Jonathan Kermanshahchi, Birpartap Thind, Gabriel Davoodpour, Megan Hirsch, Jeff Chen, Akshay J Reddy, Zeyu Yu, Benjamin E Falkenstein, Daryoush Javidi

**Affiliations:** 1 Medicine, California University of Science and Medicine, Colton, USA; 2 College of Medicine, California Health Sciences University, Clovis, USA; 3 Medical Education, California University of Science and Medicine, Colton, USA

**Keywords:** tavr versus savr, cost-effectiveness, savr, tavr, aortic valve, length of stay, complications, comorbidities

## Abstract

This review provides an in-depth analysis of the effect of length of stay (LOS), comorbidities, and procedural complications on the cost-effectiveness of transcatheter aortic valve replacement (TAVR) in comparison to surgical aortic valve replacement (SAVR). We found that the average LOS was shorter for patients undergoing TAVR, contributing to lower average costs associated with the procedure, although the LOS varied between patients due to the severity of illness and comorbidities present. TAVR has also been found to improve the quality of life for patients receiving aortic valve replacement compared to SAVR. Although TAVR has a lower rate of most post-operative complications caused by SAVR, such as bleeding and cardiac complications, TAVR shows an increased rate of permanent pacemaker (PPM) implantation due to mechanical trauma on the heart’s conduction system. In addition, our findings suggest that the cost-effectiveness of each procedure varies based on the types of valve, the patient history of other medical conditions, and the procedural methods. Our findings show that TAVR is preferred over SAVR in terms of cost-effectiveness across a variety of patients with other coexisting medical conditions, including cancer, advanced kidney disease, cirrhosis, diabetes mellitus, and bundle branch block. TAVR also appears to be superior to SAVR with fewer post-operative complications. However, TAVR appears to have a higher rate of PPM implantation rates as compared to SAVR. The comorbidities of the valve recipient must be considered when deciding whether to use TAVR or SAVR as cost-effectiveness varies with the patient background.

## Introduction and background

Valvular heart disease is a leading cause of cardiac-related mortality in both the male and female adult populations [1]. Aortic valve stenotic disease is the most prevalent valvular pathology in developing nations, affecting nine million people worldwide [1]. The two most prominent interventions to treat aortic valve pathology are transcatheter aortic valve replacement (TAVR) and surgical aortic valve replacement (SAVR). TAVR is less invasive than SAVR and therefore was originally used for surgical high-risk patients beginning in 2011 [2]. The literature also shows TAVR having good long-term results for high-risk patients [3], making TAVR a marvel in the progressions of aortic valve therapeutics.

In our review, we included all risk groups, varying comorbidities, and varying procedural methods. The cost of TAVR has been studied in the context of high-risk or specific comorbidity populations [4,5]; this paper provides a comprehensive overview of broader patient populations’ outcomes and costs with TAVR in comparison to SAVR specifically analyzing patients with comorbidities. TAVR was introduced to clinical practice for high-risk patients in 2011 due to its less invasive nature [2]. The cost of the procedure has changed rapidly, and we sought to understand the cost-effectiveness of TAVR versus SAVR for aortic stenosis patients while considering the length of stay (LOS), cost effects of varying comorbidities, and complications of the procedures.

## Review

Methods

TAVR emerged as a less invasive alternative to SAVR, initially reserved for high-risk surgical patients since 2011. Our comprehensive investigation encompasses diverse risk profiles, varying comorbidities, and procedural methodologies. This study is a continuation of "A Review of the Cost Effectiveness of Transcatheter Aortic Valve Replacement (TAVR) Versus Surgical Aortic Valve Replacement (SAVR)," which is a review comparing the cost of TAVR and SAVR [6]. A systematic examination was conducted from September 2022 to November 2022 in order to find the latest literature over the past 10 years by utilizing PubMed as the primary data source. The search query "TAVR and costs" yielded 231 publications within the last decade. Subsequently, a meticulous screening process was employed to adhere to inclusion criteria, which encompassed North American studies, those with patient data, comparisons between TAVR and SAVR, and discussions on LOS, patient comorbidities, or complications. Non-North American studies were excluded.

**Figure 1:**
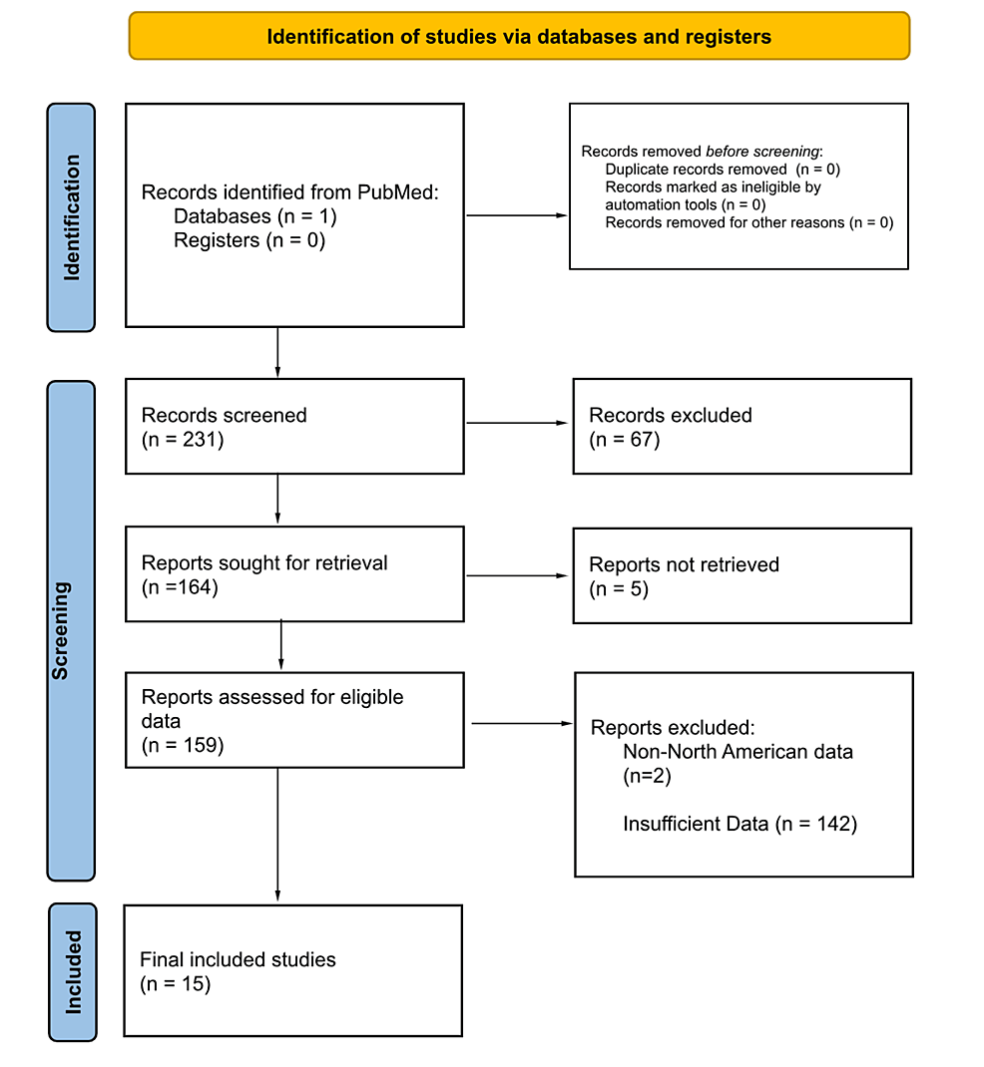
PRISMA diagram for reviewed studies PRISMA: Preferred Reporting Items for Systematic Reviews and Meta-Analyses

The assessment of cost-effectiveness incorporated factors, such as LOS, cost of index hospitalization and procedure, quality of life years (QALYs) gained, post-procedural follow-up costs, complication rate, and mortality rate. Out of the 231 initially identified publications, 67 were excluded due to non-compliance with inclusion criteria. Five were omitted due to unattainable access to the reports, while two were dismissed as the data source did not originate from North America. Another 142 studies were excluded from the review due to insufficient data for the prescribed analysis. Ultimately, 15 studies met the stringent criteria for inclusion in our analysis. The results of this selection process are displayed in Figure 1.

LOS differences between TAVR and SAVR

One of the main reasons for TAVR’s increase in usage (as an alternative to SAVR) is its shorter LOS at the hospital. As such, certain complications are more so correlated with increased LOS than others. One study found three factors to be associated with increased LOS: history of prior atrial fibrillation (p < 0.001), each one-year increase in patient age (p < 0.001), and patient urgency as indicated by pre-TAVR admission of three or more days (0.009) [7].

In 2012, the mean LOS for TF-TAVR (transfemoral-TAVR, which is the typical approach) was found to be six days shorter than that for SAVR and had a mean difference of >2 intensive care unit days, largely due to lower non-procedural costs [8]. Four years later, it was found that TAVR reduced the LOS by an average of 4.4 days, decreased the need for rehabilitation post-procedure, and resulted in a better one-month quality of life when compared to SAVR [9].

In a 2018 comparative study looking at Blue Cross Blue Shield of Michigan and Medicare fee-for-service beneficiaries undergoing TAVR or SAVR, it was again found that TAVR’s mean hospital LOS was shorter, at 6.2 ± 5.6 days [10]. Meanwhile, SAVR’s mean LOS measured 10.2 + 7.5 days [10]. Similarly, a 2016 propensity-matched cost analysis of TAVR versus SAVR found that the post-operative LOS was six days for patients receiving TAVR, seven days for intermediate-risk patients receiving SAVR, and eight days for high-risk patients receiving SAVR [11]. In fact, this study also found that the ICU LOS was shorter for TAVR patients, too, with an average of 48 hours, compared with 56 hours for intermediate-risk SAVR and 70 hours for high-risk SAVR [11]. A 2020 study confirmed this finding, stating that TAVR (when performed with percutaneous coronary intervention) had “significantly shorter (median) LOS” at six days, compared with SAVR’s eight days (SAVR was performed with coronary artery bypass grafting) [12].

LOS is often influenced by adverse events following surgery, and given that being female is a risk factor for increased adverse events during cardiac surgery, it was important to examine the LOS for female TAVR recipients [13]. One study looked at female patients who either underwent TAVR or SAVR from 2011 to 2014 in the Healthcare Cost and Utilization Project's National Inpatient Sample (NIS) database (n = 21,661) and found that the LOS for female patients who underwent TAVR was shorter (7.8 days) compared to patients who received SAVR (10.5 days) [13]. Interestingly, patients who received TAVR during this time period were in general older and had a higher burden of comorbidities than SAVR recipients [13].

Procedural methods also impact the LOS associated with TAVR [14,15]. One study found that using XT-TAVR versus SAVR reduced the total hospital stay by 4.5 days and ICU stay by 2.2 days [14]. Another finding in the study showed that S3-TAVR reduced the total LOS by 6.3 days total and 2.8 days less spent in the ICU compared with SAVR [14]. Due to the shortened lengths of time, non-procedural costs associated with index hospitalization were $25,413 lower with XT-TAVR than SAVR and lower with S3-TAVR $27,119 than SAVR [14]. Similarly, a 2021 marginal analysis completed at the Mayo Clinic analyzed the cost-effectiveness of TAVR in relation to the post-procedural LOS in the hospital [15]. The use of minimally invasive TAVR was compared to traditional TAVR and SAVR. It was found that the LOS at the hospital after TAVR was three to seven days, as opposed to SAVR’s four to eight days [15]. Percutaneous femoral artery access was also found to result in shorter hospital days by one to three days in comparison to femoral surgical cutdowns, with no difference in vascular events or mortality at 30 days [15]. Therefore, it is concluded that the minimally invasive TAVR has reduced LOS compared with traditional TAVR and SAVR practice [14,15].

TAVR’s shorter LOS is also “continuing to shorten” over time [15]. In fact, in a 2019 economic study of the PARTNER 2 clinical trials (for patients with severe aortic stenosis at intermediate surgical risk), Baron and colleagues stated that “the higher procedure costs associated with TAVR were offset by significant reductions in other costs, driven primarily by reductions in the total LOS” [14]. Kumar and colleagues stated that this reduction in LOS is largely driven by the switch from intubation to conscious sedation, which reduces hospital stay [15]. Furthermore, a 2020 study evaluating varying temporal trends of TAVR found that the LOS decreased from a median of seven to two days (p < 0.001) between January 1, 2012, and December 31, 2017 [16]. This study presented that over time, there has been a decrease in resource use, cost, and burden of hospitalizations for patients that undergo TAVR, but it also found an increase in expenditure on TAVR-related hospitalizations [16].

**Figure 2:**
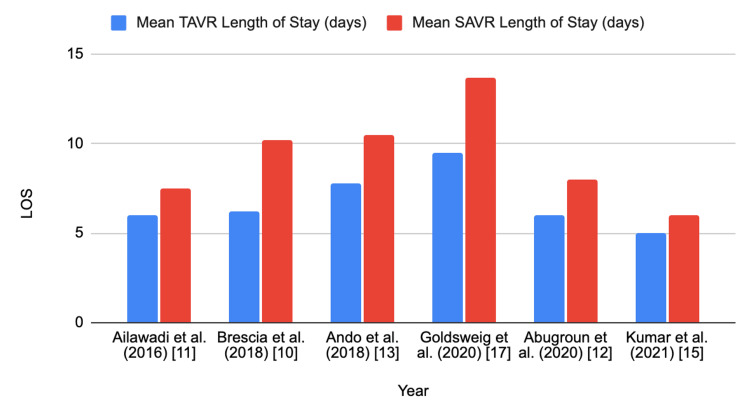
Mean TAVR versus SAVR LOS LOS: length of stay, TAVR: transcatheter aortic valve replacement, SAVR: surgical aortic valve replacement

Despite the shorter index LOS in TAVR recipients, readmission rates are higher [10,16,17]. A 2017 study on 30-day readmission rates after TAVR found that 17.9% of patients were readmitted within 30 days, with an index hospitalization length of greater than five days as a predictor for readmission [16]. Readmissions added an average of four days to the total LOS [16]. As Table 1 showcases, Goldsweig and colleagues found that TAVR recipients experienced shorter LOS irrespective of risk group, ranging from five to 14 days, depending on the risk group and comorbidities; that of SAVR ranged from seven days to 20.3 days [17]. However, they also found that the unplanned readmission inpatient days were higher with TAVR (3.0) versus SAVR (2.0), with the average number of total admissions for TAVR being 1.5 and 1.3 for SAVR [17]. However, it is to be noted that in this retrospective study (n = 190,563), TAVR was performed in intermediate to greater surgical risk, which may help explain the trend in readmission [17]. In a 2018 study, readmission rates were similar between TAVR (23.1%) and SAVR (22.7%), but readmission payments were almost 25% lower for TAVR [10]. Figure 2 showcases the mean LOS of TAVR versus SAVR.

**Table 1:** Length of stay differences between TAVR and SAVR TAVR: transcatheter aortic valve replacement, SAVR: surgical aortic valve replacement, TF: transfemoral

Authors (year)	Year	TAVR length of stay (mean, unless stated otherwise)	SAVR length of stay (mean, unless stated otherwise)	Notes
Kumar et al. (2021) [15]	2021	3-7 days	4-8 days	
Abugroun et al. (2020) [12]	2020	6 days (median)	8 days (median)	
Goldsweig et al. (2020) [17]	2020	5-14 days	7-20.3 days	Ranges are based on risk group and comorbidities
Baron et al. (2019) [14]	2019	4.5 days shorter than SAVR	–	XT-TAVR TAVR ICU: 2.2 days shorter than SAVR
Ando et al. (2018) [13]	2018	7.8 days	10.5 days	
Brescia et al. (2018) [10]	2018	6.2 ± 5.6 days	10.2 + 7.5 days	
Ailawadi et al. (2016) [11]	2016	6 days	7-8 days	TAVR ICU: 48 hours SAVR ICU: 56-70 hours
Reynolds et al. (2016) [9]	2016	4.4 days shorter than SAVR	–	
Reynolds et al. (2012) [8]	2012	6 days shorter than SAVR	–	TF-TAVR TAVR ICU: >2 days shorter than SAVR

How cost and outcomes are affected by comorbidities

In order to best assess the most cost-effective procedure for a patient, we must compare the cost associations between TAVR and SAVR in the context of analyzing the costs associated with different comorbidities. In a 2022 study of Medicare beneficiaries, it was found that of the intermediate-risk TAVR recipients, 92.1% had hypertension, 39.0% had diabetes, 61.0% had peripheral vascular disease, and 30.1% had renal failure [4]. SAVR recipients had similar rates of comorbidities, and high-risk recipients of TAVR and SAVR had generally higher rates of comorbidities. Patients with a higher Elixhauser comorbidity index, a method of categorizing comorbidities of patients based on the International Classification of Diseases (ICD) diagnosis codes found in administrative data, were more likely to receive TAVR than SAVR [4].

Patients who are diagnosed with advanced kidney disease (AKD), such as chronic kidney disease (CKD) and end-stage renal disease (ESRD), may have this particular comorbidity affect their outcomes when being treated with TAVR and SAVR [18]. A 2017 retrospective study using a sample from the NIS between 2012 and 2014 was done to discern the differences in outcomes between AKD patients receiving TAVR or SAVR [18]. The mortality rate was lower for TAVR as compared to SAVR (6.2% and 12.9%, respectively) [18]. As Table 2 demonstrates, the median LOS was also lower for TAVR as compared to SAVR (eight and 14 days, respectively) [18]. Furthermore, the mean cost of hospitalization was also lower for TAVR as compared to SAVR ($58,927 and $62,295, respectively) [18]. However, it was also found that the permanent pacemaker (PPM) placement rate was higher in patients undergoing TAVR than those receiving SAVR (27.8% and 9.3%, respectively) [18]. TAVR is found to benefit patients with AKD, and it can be deemed as a safe and cost-effective alternative to high-risk patients who cannot undergo surgery [18].

**Table 2:** TAVR versus SAVR cost and LOS comparison by comorbidity LOS: length of stay, ESRD: end-stage renal disease, AKD: advanced kidney disease, CKD: chronic kidney disease, COPD: chronic obstructive pulmonary disease

Authors (year)	Year	Comorbidity	TAVR cost	SAVR cost	TAVR LOS	SAVR LOS
Monlezun et al. (2021) [23]	2021	Cancer	$216,458.70	$242,302.10	5.16 ± 6.04 days	10.19 ± 9.51 days
Khan et al. (2020) [19]	2020	ESRD	$311,538.16 to $255,693.40; p	$402,019.11 to $466,567.00; p	–	–
Ando et al. (2017) [22]	2017	Diabetes mellitus	$71,960	$78,060	6 days	8 days
Dhoble et al. (2018) [21]	2017	Cirrhosis	$98,617	$77,640	6.3 days	10.2 days
Doshi et al. (2017) [18]	2017	AKD/CKD/ESRD	Mean cost of hospitalization: $58,927	$62,295	8 days	14 days
Abugroun et al. (2021) [20]	2021	COPD	$53,255	$42,396	–	–

In addition to Doshi’s study, Khan and colleagues used the NIS to study trends in patients with ESRD undergoing TAVR and SAVR (January 2012 and December 2017) [19]. In this six-year study of in‐hospital trends in ESRD patients undergoing aortic valve replacement, researchers found that although patients undergoing TAVR have significantly higher comorbidity burden, inpatient mortality declined over time with TAVR but not with SAVR while also having considerably lower complications than SAVR [19]. Furthermore, in the study population, TAVR was associated with reduced LOS and cost of care, as well as a higher proportion of discharge to home when compared with SAVR [19]. This is important because ESRD patients are known to have significantly higher risk of vascular complications for several reasons, such as bleeding diathesis and suboptimal vascular access, which can occur due to stenosis and calcifications; SAVR has shown to lead to higher rates of bleeding and blood transfusion requirements in patients compared to TAVR [19]. The cost of stay among the ESRD TAVR cohort (2012-2017) was $311,538.16 to $255,693.40 (p < .001) compared to SAVR subjects ($402,019.11 to $466,567.00; p < .001) [19].

Transitioning from ESRD to chronic obstructive pulmonary disease (COPD), a study looking at the outcomes of transapical versus surgical aortic valve replacement from the US NIS queried for all patients who underwent TA-TAVR or SAVR during the years 2016−2017 found that TA-TAVR correlated with a higher mortality risk among patients with COPD but similar mortality rates in subgroups including patients ≤75 years, females, and those with chronic kidney disease when this study was compared to a similar one from Germany [20]. The cost of hospitalization with TA-TAVR was $53,255 (IQR: 39,028−73,295) and that of SAVR was $42,396 USD (IQR: 32,564−59,339) [20].

Patients with cirrhosis are particularly at a higher risk of mortality (5.7%) and early readmission (20%) after aortic valve replacement procedures [21]. A study analyzing the NIS and National Readmission Database (NRD) analyzed the difference between patients with cirrhosis who have undergone TAVR versus SAVR [21]. This study found that “there were no significant differences between the in-hospital mortality, readmissions, hospitalization costs, and discharges to home within the TAVR and SAVR groups” [21]. However, the TAVR post-procedure LOS was significantly lower than SAVR (6.3 vs. 10.2 days; p < 0.001) with a significantly lower blood transfusion rate (22% vs. 58%; p < 0.001) [21]. This translates to a difference in the total hospital cost from the two procedures [21]. The total hospital cost of TAVR is displayed as $98,617, while the total hospital cost of SAVR was found to be $77,640 [21].

Diabetes mellitus (DM) is widespread in patients, which makes it crucial to understand the post-procedural costs and outcomes associated with TAVR versus SAVR in patients with DM [22]. A study analyzing 5,719 TAVR procedures and 65,096 SAVR procedures from 2011 to 2013 found that TAVR patients were at lower risk for bleeding requiring transfusions (13% vs. 20%, OR 0.43, p < 0.01), cardiac complications (6.1% vs. 14%, OR 0.34, p < 0.01), and post-operative sepsis (1.7% vs. 3.6%, OR 0.45, p = 0.03) as compared to SAVR [22]. However, TAVR patients were at an increased risk for pacemaker implantations (10% vs. 5.7%, OR 1.5, p < 0.01) [22]. In terms of the mean hospitalization cost, TAVR was lower than SAVR with ($71,960 vs. $78,060, p = 0.003) [22]. The LOS of TAVR was also significantly lower than that of SAVR (median six vs. eight days, p < 0.001), which contributes to the overall lower cost as well [22].

Oftentimes, patients with cancer who are in need of an aortic valve replacement, such as TAVR or SAVR, are excluded from obtaining the procedure [23]. Having cancer can influence the clinical and cost outcomes of patients undergoing TAVR and SAVR [23]. In a 2021 population-based case-control study of TAVR being done in patients who are also diagnosed with cancer, again, the LOS for TAVR (mean 5.16 ± 6.04 days) was significantly shorter than the LOS for SAVR (mean 10.19 ± 9.51 days) [23]. Furthermore, in patients with cancer, TAVR resulted in a lower mortality rate as compared to SAVR (1.43% vs. 3.24%, respectively) [23]. The mortality rate in patients who underwent TAVR was also statistically similar in both the cancer and non-cancer groups (1.43% vs. 1.98%) [23]. Whether the patient had cancer or not had no effect on the mortality rate of the TAVR procedure [23]. In terms of cost, TAVR was lower with a mean USD $216,458.70 (SD 136,223.5) compared to SAVR with $242,302.10 (SD 232,242.80) [23]. The benefits of TAVR are clear in both clinical and cost outcomes among patients who are comorbid with cancer compared to the alternative of SAVR [23]. Furthermore, it was found that TAVR has similar outcomes in patients co-existing with cancer and those who do not have cancer [23]. TAVR should not be denied to those co-existing with cancer [23]. 

Another comorbidity studied was a bundle branch block [24]. Patients with a left bundle branch block were seen to have very little effect on their cost of TAVR, with $47,552 (compared to $47,171 with no bundle branch block) [24]. However, patients with the right bundle branch block saw a significant increase in cost, at $53,669 [24]. It is also important to recognize the effects of various conditions on the average LOS after TAVR [24]. For example, the LOS for TAVR patients with a right bundle branch block is significantly longer than those with left or no bundle branch block, at five days, three days, and three days, respectively [24].

In addition to bundle branch block, cardiac amyloidosis should also be considered with patients undergoing aortic valve replacement. Khan’s study compared TAVR and SAVR in patients with cardiac amyloidosis [25]. The presence of cardiac amyloidosis is shown to have a significantly worse outcome than those without cardiac amyloidosis [25]. Previous studies have shown that TAVR yielded a better outcome in aortic stenosis-cardiac amyloidosis patients than medical therapy [25]. This study shows, after adjustments for confounding factors, that the SAVR group has higher mortality and cost, while the LOS was shorter for the TAVR group, as we may have predicted [25]. TAVR and SAVR each yield slightly different rates for complications [25]. Patients in the TAVR group are more likely to develop stroke, vascular complication, and permanent pacemaker implantation, while the patients in the SAVR group were more prone to develop acute myocardial infarction, acute kidney injury, and major bleeding, which is reasonable due to the more invasive nature of SAVR [25].

TAVR has also been found to be an excellent option for inoperable or very high-risk aortic stenosis patients; a 2012 study focused on the QALYs gained, cost, and outcomes for this patient population [8]. They found that among patients eligible for transfemoral TAVR, they saved an average of $1,250 and had a “modest gain” in QALYs, when compared to SAVR [8]. However, for patients that required a transapical approach to TAVR, the opposite was true, demonstrating the importance of studying the TAVR procedural methods when looking at both outcomes and costs [8].

Comparing the complications associated with TAVR versus SAVR

In terms of complications, studies have shown varying results. However, generally, both techniques can present various complications, but some are more prevalent in one than the other. Both TAVR and SAVR are associated with complications of stroke, cardiac arrest, vascular complications, blood transfusions, and cardiogenic shock [26]. In particular, TAVR generally showcases lower incidences of complications, such as strokes, cardiogenic shock, acute kidney injury, and blood transfusion [26], but higher incidences of permanent pacemaker implantation, cardiac arrest, and vascular complications [26].

For instance, Abugroun et al. showed that after risk adjustment, both TA-TAVR and SAVR became similar in terms of mortality, risk of acute kidney injury, periprocedural cardiac complication rates, complications such as stroke, need for pacemaker insertion, vascular complications, and mechanical ventilation [20]. However, TA-TAVR was correlated with statistically significant higher rates of pericardiocentesis (0.6% vs. 0.3%, SMD = 0.1) and acute kidney injury (25.3% vs. 18.8%, SMD = 0.2) but lower rates of major bleeding (4.8% vs. 11.4%, SMD = −0.2) compared to SAVR [20].

It is also important to consider the risk of permanent pacemaker implantation when considering post-operative risk assessments for TAVR versus SAVR [27]. TAVR has historically had a high rate of permanent pacemaker implantation in comparison to SAVR [27]. The prevalence of permanent pacemaker implantation after TAVR ranges from 9% to 26% (Ruck), which can be explained by the positioning of the aortic valve annulus with respect to the cardiac conduction system [27]. In a previous study analyzing intermediate-risk AS patients who were treated with TAVR or SAVR in the PARTNER 2 trial, it was found that permanent pacemaker placement was slightly higher in those patients with either S3-TAVR or XT-TAVR in comparison to SAVR (72 vs. 66, p = 0.829; 90 vs. 66, p = 0.251), respectively [4].

The trend seems to be similar when looking at patients with comorbidities, such as ESRD [28]. In a study comparing characteristics of TAVR and SAVR for patients that are on dialysis, it has been found that patients who underwent TAVR had more permanent pacemaker implantation (13.2% vs. 5.6%, p = 0.012) [28]. However, the same study concluded that TAVR is associated with lower hospital mortality, resource utilization, and cost in comparison to SAVR [28]. Although TAVR appears to have more associated permanent pacemaker implantations, it is overall associated with less post-procedural complications, such as bleeding [28].

Furthermore, post-operative delirium is often seen in older patients who undergo an operation/surgery and is one of the most common post-operative complications for older patients [29]. The patient population for those who are most in need of the treatments of both SAVR and TAVR are usually older adults, so it is imperative to look into post-operative delirium’s effect on cost and outcomes [29]. It is already known that post-operative delirium is associated with worse long-term survival in both SAVR and TAVR, but its effect on resource utilization is still in question [29]. In a 2019 retrospective comparative study, it was found that delirium was associated with a 4.16 +/- 0.65 increase in hospital LOS and a $15,592 +/- 2,743 increase in incremental hospital cost on average for both TAVR and SAVR [29]. However, it is important to note that fewer TAVR patients were affected by post-operative delirium compared to SAVR patients (1.6% vs. 3.6%, respectively) [29]. Therefore, adjusted for each treatment, TAVR resulted in +$13,862 +/- 4,431 incremental hospital cost and +3.39 +/- 1.05 LOS, while SAVR resulted in +$16,656 +/- 3,479 incremental hospital cost and +4.63 +/- 0.82 LOS [29]. In another retrospective observational study using the NIS, it was found that post-operative delirium in TAVR patients increased cost by 31.5% and increased LOS by 70.3% [30]. Overall, both SAVR and TAVR post-operative deliriums lead to significant increases in cost and hospital LOS [29,30]. However, post-operative delirium in TAVR patients does lead to less increases in both cost and LOS when compared to the surgical alternative in SAVR patients [29,30]. 

Current limitations

This study possesses certain limitations that warrant consideration. First, the reliance on data primarily sourced from PubMed may result in a potential limitation by constraining the data pool, potentially overlooking pertinent studies available in other databases or publications. This exclusive reliance may not encompass the entirety of relevant research on the subject. In addition, the study's inclusive approach to various risk groups, comorbidities, and procedural methods introduces complexity in making direct comparisons between TAVR and SAVR. The heterogeneity within patient populations may introduce confounding variables that could affect the precision of comparisons. The assessment of costs associated with TAVR is based on a comprehensive overview of broader patient populations, specifically focusing on individuals with comorbidities undergoing the procedure. However, it is essential to acknowledge the dynamic nature of healthcare costs, and the lack of standardized cost reporting across studies may influence the accuracy of cost-related comparisons. The study's time frame for the literature search is limited, spanning from 2011 to the present, and the exclusion of non-English studies may result in overlooking recent and global insights. The omission of long-term follow-up data and the exclusion of non-published data could introduce bias and limit the study's ability to draw comprehensive conclusions. Furthermore, the study does not provide an in-depth breakdown of costs, and there may be a potential oversight of healthcare system variations or changing market dynamics that could impact TAVR costs over time. Despite the valuable insights provided, researchers should be cognizant of these limitations and address them in future studies to enhance the understanding of the cost-effectiveness of TAVR and SAVR.

## Conclusions

TAVR’s LOS has been consistently shorter than SAVR’s, even for patients with varying comorbidities and complications; this shorter LOS contributes to the cost-effectiveness of TAVR. Specifically, patients with cancer, diabetes, cirrhosis, and AKD were found to have shorter LOS with TAVR. However, patients with a right bundle branch block had significantly longer LOSs with TAVR. In addition, TAVR appears to be superior to SAVR with fewer post-operative complications. However, TAVR appears to have a higher rate of PPM implantation rate as compared to SAVR. The patient comorbidities must be considered as TAVR was found to have a lower cost for patients with cancer, ESRD, diabetes, and advanced kidney disease, but a higher cost for patients with cirrhosis and COPD. The review of TAVR versus SAVR cost ("A Review of the Cost Effectiveness of Transcatheter Aortic Valve Replacement (TAVR) Versus Surgical Aortic Valve Replacement (SAVR)") came to the conclusion in which new TAVR valves entering the market are allowing the total procedural cost of TAVR to decrease over time compared to SAVR. This review found that TAVR has a lower cost on patients with certain comorbidities in addition to a lower LOS than SAVR. This information can help tailor clinical decisions based on the patient’s unique comorbidity and history in order to provide better outcomes and cost-saving measures to treat aortic valve pathology.
